# Major James Carroll

**Published:** 1907-10

**Authors:** 


Major James Carroll, M.D., Surgeon United States Army, died at Washington, September 16, 1907, aged 53 years. He was the third member of the original Cuban yellow fever commission to die, one only remaining, Dr. Agramonte. At the time of his death Surgeon Carroll was curator of the Army Medical Museum and professor of bacteriology and clinical microscopy in the Army Medical School.

